# Smile Mimicry and Emotional Contagion in Audio-Visual Computer-Mediated Communication

**DOI:** 10.3389/fpsyg.2018.02077

**Published:** 2018-11-05

**Authors:** Phoebe H. C. Mui, Martijn B. Goudbeek, Camiel Roex, Wout Spierts, Marc G. J. Swerts

**Affiliations:** Department of Communication and Cognition, Tilburg University, Tilburg, Netherlands

**Keywords:** smile mimicry, emotional contagion, computer-mediated communication, audio-visual communication, nonverbal behavior

## Abstract

We investigate whether smile mimicry and emotional contagion are evident in non-text-based computer-mediated communication (CMC). Via an ostensibly real-time audio-visual CMC platform, participants interacted with a confederate who either smiled radiantly or displayed a neutral expression throughout the interaction. Automatic analyses of expressions displayed by participants indicated that smile mimicry was at play: A higher level of activation of the facial muscle that characterizes genuine smiles was observed among participants who interacted with the smiling confederate than among participants who interacted with the unexpressive confederate. However, there was no difference in the self-reported level of joviality between participants in the two conditions. Our findings demonstrate that people mimic smiles in audio-visual CMC, but that even though the diffusion of emotions has been documented in text-based CMC in previous studies, we find no convincing support for the phenomenon of emotional contagion in non-text-based CMC.

## Introduction

Encountering a happy person who smiles radiantly may make you smile too and, subsequently, lift your mood as well. These phenomena of *smile mimicry* and *emotional contagion* have been documented in numerous studies; emotional contagion is theorized as a multi-stage mechanism that comprises mimicry (Hatfield et al., 1992, 1993, 2014). Despite their social nature, smile mimicry and emotional contagion have been investigated mostly in artificial laboratory settings devoid of a social and communicative element, or in text-based computer-mediated communication (CMC) in which facial mimicry cannot be ascertained due to the invisibility of the face. Even in recent CMC studies on emotions (Holtzman et al., 2017; Kafetsios et al., 2017), the specific type of CMC investigated was either text-based only or unspecified. In other words, it is still unclear to what extent smile mimicry and emotional contagion are at play in non-text-based audio-visual CMC channels, which afford users rich information on par with that available in face-to-face interactions but is absent in text-based channels. Thus, we addressed the following research questions in this study: Do people mimic smiles in audio-visual CMC? If so, is the mimicry of smiles accompanied by enhanced positive affect, indicative of emotional contagion?

### Smile Mimicry and Emotional Contagion

People tend to imitate the verbal and non-verbal behavior of their interaction partner (Chartrand and Bargh, 1999; Lakin and Chartrand, 2003; Chartrand and Lakin, 2013). The tendency to mimic other people’s behavior appears to be automatic, non-conscious, and difficult to suppress (Dimberg et al., 2000; Korb et al., 2010). Given the prominence of the face, facial expressions are particularly susceptible to mimicking (Chartrand et al., 2005). Indeed, there is considerable evidence for smile mimicry, which is a form of facial mimicry. Participants who viewed happy stimuli displayed increased activity in their *zygomaticus major*, the muscle that pulls lip corners up and produces a smile (e.g., Dimberg and Thunberg, 1998; Dimberg et al., 2000). Some researchers (e.g., Hess and Bourgeois, 2010; Heerey and Crossley, 2013; Krumhuber et al., 2014) have differentiated between the mimicry of two types of smiles: smiles activated by only the *zygomaticus major* (non-Duchenne smiles), and smiles characterized by activity in both the *zygomaticus major* and the *orbicularis oculi*, which is the muscle that orbits the eyes (Duchenne de Boulogne, 1990). Such Duchenne smiles have long been regarded as spontaneous smiles that reflect felt positive affect, whereas non-Duchenne smiles have been regarded as deliberate smiles displayed in the absence of positive affect (Ekman and Friesen, 1982; Frank and Ekman, 1993; but see e.g., Gunnery et al., 2013).

In face-to-face interactions, when people mimic the facial expressions of others, they may also come to experience the emotions underlying the expressions they mimic, a phenomenon referred to as *emotional contagion* (Hatfield et al., 1992, 1993, 2014). Emotional contagion is defined as “the tendency to mimic and synchronize the movements, expressions, postures, and vocalizations with those of another person and, consequently, to converge emotionally” (Hatfield et al., 1992, pp. 153–154). Emotional contagion is assumed to be a multi-stage mechanism comprising mimicry and afferent feedback: When one perceives an emotional expression displayed by others, one spontaneously mimics that expression, which provides afferent feedback to oneself, eliciting in oneself the same emotion as the one experienced by others (e.g., Dimberg, 1988; Hatfield et al., 1992, 1993, 2014).

Even though mimicry is social in nature, the majority of studies on mimicry have relied on electromyography (EMG), in which participants were passively exposed to emotional stimuli in a lab (e.g., Dimberg and Lundqvist, 1990; Dimberg and Thunberg, 1998; Dimberg et al., 2000). With few exceptions (e.g., Mojzisch et al., 2006; Krumhuber et al., 2014), the emotional stimuli in question have been static or synthetic. In other studies, participants were videotaped while they were presented with similar stimuli; their expressions were coded afterward (e.g., Hinsz and Tomhave, 1991; Sato and Yoshikawa, 2007), sometimes in accordance with the facial action coding system (FACS; Ekman and Friesen, 1978). However, passively viewing successive emotional stimuli hardly resembles how people typically engage with others in their social interactions, especially when the stimuli in question are static. Also, due to the absence of a communicative element, not only is the ecological validity of such studies limited, but the social nature of mimicry is overlooked as well. A few researchers have enhanced the social nature or relevance of stimuli by testing participants in dyads and manipulating their relative status (Hess and Bourgeois, 2010), or by presenting smiles to participants giving correct responses in a task (Heerey and Crossley, 2013). Still, having electrodes attached to one’s skin or being aware that one is being videotaped is hardly conducive to a naturalistic experience. Thus, there remains a need to study the occurrence of mimicry and emotional contagion in more naturalistic social settings.

Some attempts have been made to investigate the diffusion of emotions in actual communicative settings, albeit mostly in text-based CMC. Coviello et al. (2014) have investigated whether the emotional content of text-based status updates posted by users of Facebook would also influence the status updates of friends of those users; status updates were classified as reflecting a positive emotion or a negative emotion, as determined by the Linguistic Inquiry Word Count (Tausczik and Pennebaker, 2010). Adopting a similar but more experimental approach, Kramer et al. (2014) manipulated the news feed of users of Facebook, such that users were exposed to a reduced number of text-based positive (negative) updates. They have found that the updates subsequently posted by users reflected the content of their news feed: Updates of users exposed to fewer positive (negative) updates contained a lower percentage of positive (negative) words. Similarly, Ferrara and Yang (2015) have found that in Twitter, the emotional content of tweets posted by users was influenced by the emotional content of tweets posted by other users.

However, the phenomenon studied in these CMC studies was not consistent with the definition of emotional contagion commonly adopted in the literature. As described earlier, emotional contagion comprises mimicry (Hatfield et al., 1992, 1993, 2014). The results of the aforementioned CMC studies were based on the affective valence of text posted by users only. Therefore, non-verbal mimicry and, consequently, emotional contagion, could not be ascertained. Even though emoticons can be used as paralinguistic cues to convey affective information (Aldunate and González-Ibáñez, 2017), text-based CMC alone does not allow for an investigation of actual facial mimicry (Derks et al., 2008). In other words, it is still unclear to what extent facial mimicry and emotional contagion are at play on cue-rich, non-text-based CMC platforms such as audio-visual CMC, which afford users rich information comparable with that available in face-to-face interactions.

From a theoretical perspective, investigating smile mimicry and emotional contagion in audio-visual CMC extends our understanding of these phenomena to communication channels that have become ubiquitous in modern day life. From a methodological perspective, audio-visual CMC not only closely resembles the kind of daily social interactions many of us encounter, but also ensures a high degree of experimental control, as it allows for identical stimuli to be shown to participants assigned to the same condition (i.e., video footage can be pre-recorded, as described later in the Materials and Method section).

### Overview of Present study

We set out to investigate smile mimicry and emotional contagion in cue-rich CMC. We administered a task on what appeared to be a live video communication platform to participants. Participants were seated in front of a computer and were randomly assigned to interact with a partner who either radiated with smiles or maintained a neutral expression. Unbeknownst to the participants, the partner was a confederate, whose speech and facial expressions had been pre-recorded. In other words, in each condition, participants were “interacting” with the same video footage, not with a person real-time (as per the O-Cam paradigm; Goodacre and Zadro, 2010). Before and after the interaction, participants reported their affective states; these were analyzed to measure emotional contagion.

To examine if participants mimicked their partner’s smiles, their facial expressions displayed were videotaped and subsequently analyzed by the Computer Expression Recognition Toolbox (CERT; Littlewort et al., 2011). CERT is a fully automated facial expression recognition system, capable of real-time, frame-by-frame coding of 20 action units (AUs) and a number of prototypic facial expressions; its output has been shown to correlate with the intensity codes provided by trained FACS coders (Bartlett et al., 2006). We used CERT to code the activation of *zygomaticus major* (AU 12; lip corner puller) as well as *orbicularis oculi* (AU 6; cheek raiser), as both AU 6 and AU 12 are activated in a Duchenne smile, whereas only AU 12 is activated in a non-Duchenne smile (for previous works using CERT to code smiles, see Rychlowska et al., 2014; Ruvolo et al., 2015). We focused on mimicry of smiles and not on other non-verbal features for two reasons: First, because the face is almost always visible in audio-visual CMC, it makes sense to focus on a form of facial mimicry. Second, the existing knowledge of mimicry is largely based on smile mimicry; examining smile mimicry allows for comparisons with previous studies.

Given the evidence for smile mimicry and emotional contagion reported in non-social lab settings, as well as the evidence for the diffusion of emotions observed in text-based CMC, we expected that in an interactive audio-visual CMC:

**H1**: Participants interacting with a smiling partner would display a higher level of activation in AU 6 and AU 12 than participants interacting with a neutral partner.

**H2**: Participants interacting with a smiling partner would report to be more jovial after the CMC than before the CMC, whereas participants interacting with a neutral partner would not.

## Materials and Methods

### Participants

Fifty-four students from Tilburg University took part in the present study in exchange for partial course credit. This sample size is comparable to those in previous studies on smile mimicry (i.e., Dimberg and Lundqvist, 1990: *N* = 48; Krumhuber et al., 2014: *N* = 30) and on emotional contagion (i.e., Dimberg, 1988: *N* = 22; Lundqvist and Dimberg, 1995: *N* = 56). All participants were probed for suspicions at the end of the experiment. Two participants suspected that the interaction was not real; their data were excluded from subsequent analyses. The final sample consisted of 52 participants (40 female; female: *M*_age_ = 21.25, *SD*_age_ = 2.48; male: *M*_age_ = 22.25, *SD*_age_ = 2.38). Twenty-seven participants (20 female) interacted with a smiling partner (the *smiling condition*), and the remaining 25 (20 female) interacted with a partner whose expression was neutral (the *neutral condition*), the assignment of which was random. None of the participants reported having met their partner, who was a confederate, prior to the experiment.

### Procedure

This study was carried out in accordance with the recommendations of the Netherlands Code of Conduct for Scientific Practice, Association of Universities in the Netherlands. The protocol was approved by the Research Ethics Committee, School of Humanities and Digital Sciences, Tilburg University. All participants gave written informed consent in accordance with the Declaration of Helsinki.

Participants were seated individually in a sound-proof booth, in front of a computer equipped with a webcam, a microphone, and speakers. Under the pretext of investigating how people describe complex figures in audio-visual CMC, Experimenter 1 informed the participant that he/she was going to interact with another student in a real-time online audio-visual interface. All participants provided written informed consent, agreeing to take part in the experiment, to be videotaped, and to have their recordings and photos analyzed for academic and research purposes. Some participants also consented to having their recordings and photos published; screenshots provided in this article were only taken from participants who gave such explicit written consent. Subsequently, the participant completed a questionnaire, consisting of demographic questions and 12 items from the expanded version of the Positive and Negative Affect Schedule (PANAS-X; Watson and Clark, 1994), the details of which are provided under Measures.

#### CMC Set-Up

**FIGURE 1:**
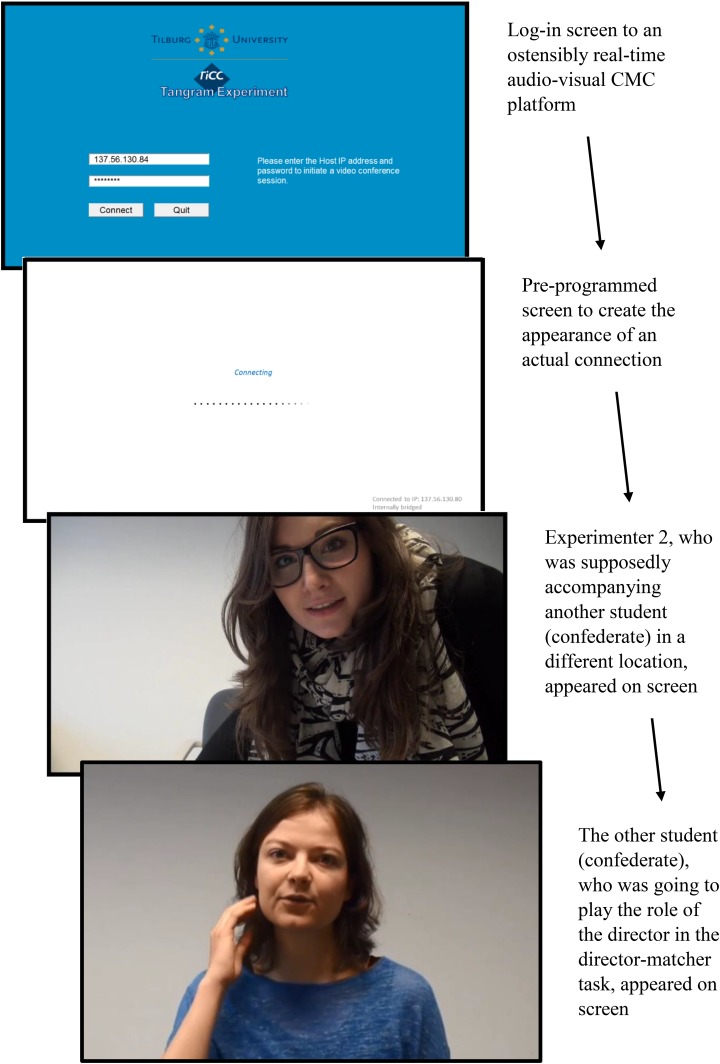
Impression of the CMC set-up, which was based on pre-recorded footage.

After the participant had completed the questionnaire, Experimenter 1 re-entered the booth and reminded the participant that the goal of the experiment was to investigate how people describe complex figures (i.e., tangram figures) in audio-visual CMC. The participant was shown examples of such figures and was informed that he/she was going to complete a director-matcher task with another student. The participant was the matcher, who had to identify target figures from given arrays, based on the descriptions given by the director. Once the participant indicated he/she was ready, Experimenter 1 switched on the webcam, entered an IP address on what appeared to be a log-in page of a video chat interface (see Figure 1 for an impression of the procedure), and waited to be connected to a web server. Shortly afterward, Experimenter 2 appeared on the screen, followed by another student (who was actually a confederate).

**FIGURE 2:**
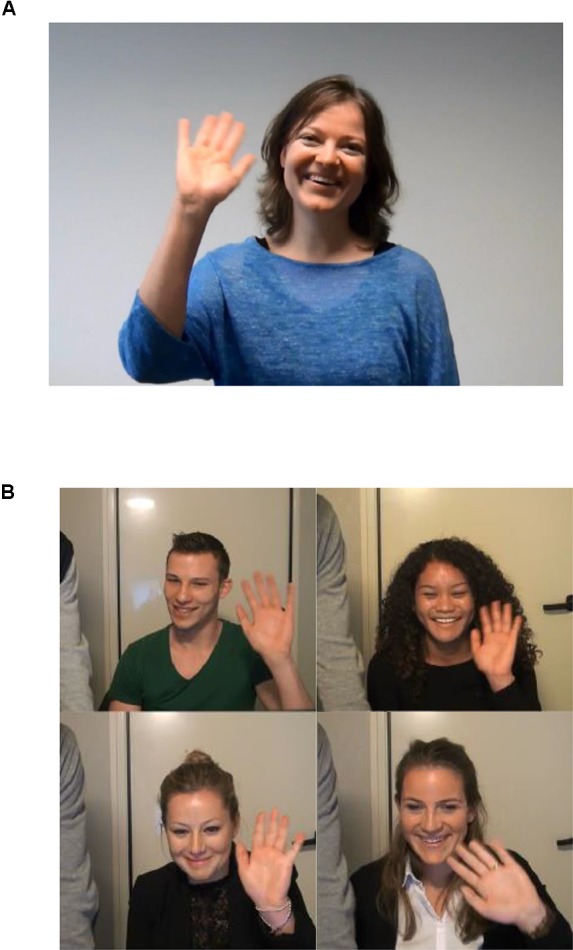
**(A)** Still of the interaction partner greeting the participant, and **(B)** example stills of participants waving back. Written consent was obtained for the publication of their identifiable images.

The two experimenters waved at each other and enquired if the volume settings were optimal. Experimenter 1 then asked the confederate to sit closer to the webcam twice so that she would be more visible on the screen. Unbeknownst to the participant, this seemingly spontaneous interaction was in fact based on pre-recorded video footage. What appeared to be a connection to a web server was simply an initialization of the footage, and the other student was actually a confederate whose actions and speech were scripted and pre-recorded. The experimenters and confederate acted at timed intervals, in order to give the impression that the interaction was live and spontaneous (as per the O-Cam paradigm; see Goodacre and Zadro, 2010). After the credibility of the interaction was established, the participant and the confederate were introduced to each other. The confederate greeted the participants and waved; in return, all participants waved back (Figure 2).

#### Director-Matcher Task

**FIGURE 3:**
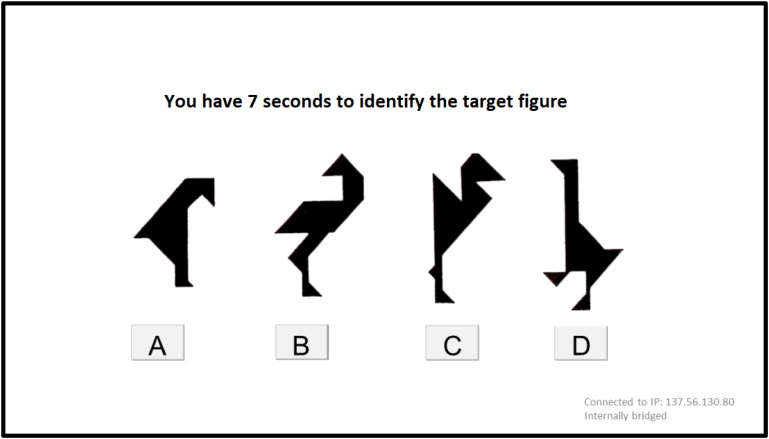
An array of tangram figures presented to participants in one of the trials.

Experimenter 2 stepped aside so that only the confederate was visible on screen; likewise, Experimenter 1 left the booth. The participant began the director-matcher task with the confederate. Four tangram figures were presented on the screen; the participant was informed that he/she had five seconds to study them. After this, a screen with the text “one moment please” was shown for four seconds, creating the impression that data was being transmitted in a real-time connection. The confederate then re-appeared and started describing the target figure. After the description, a new screen appeared, showing the same array of four figures presented earlier (see Figure 3), allowing the participant seven seconds to click on the described figure. As soon as a figure was chosen or the seven seconds had elapsed, a new similar trial began. In total, 10 trials were presented.

After the tenth trial, the participant filled out the same PANAS-X items as the ones administered before the CMC. Participants were then interviewed regarding the interaction; two participants doubted if the CMC was real, whose data were therefore excluded from analyses. All participants were debriefed fully and asked to sign a document agreeing not to disclose the true intent of the experiment to other students before the conclusion of the study.

### Smiling Condition vs. Neutral Condition

The same confederate played the role of the director in the smiling condition and in the neutral condition. The differences between the conditions surfaced in the director-matcher task: The confederate was either beaming with smiles or maintaining a neutral expression. Thus, the only essential difference was found in the facial expressions displayed by the confederate; all arrays and targets in the two conditions were identical. However, during the phase in which the confederate and the participant waved to each other (the *introduction phase*), the confederate did *not* display different facial expressions for the two conditions. This was intended to elicit a baseline expression from the participant: Subsequent differences in expressions displayed by participants could be attributed to the corresponding differences in the expressions of the confederate, and not to individual differences in expressive behavior.

### Measures

Participants were videotaped the moment the webcam was switched on (i.e., right before Experimenter 1 ostensibly logged on to the video chat platform). Segments of the footage were analyzed with CERT, which provided information regarding activity in AU 6 and AU 12. These measures allowed for an investigation of mimicry.

Participants filled out selected items of the PANAS-X (Watson and Clark, 1994) twice, once before the CMC and once after. As the present study concerned mimicry of smiles and contagion of the corresponding emotion, items concerning *joviality* (eight items; e.g., “I feel delighted right now”) were presented to participants. Internal consistency of this scale was high (pre-CMC: Cronbach’s α = 0.89; post-CMC: Cronbach’s α = 0.86). Items concerning *attentiveness* (four items; e.g., “I feel alert right now”; pre-CMC: Cronbach’s α = 0.75; post-CMC: Cronbach’s α = 0.89) were included as fillers and control variables. Participants indicated their responses on a 7-point scale (1 = *very slightly or not at all*, 7 = *extremely*).

## Results

### Validation of Stimuli

To ensure that the confederate indeed displayed smiles and came across as happy in the smiling condition, and that she came across as relatively neutral in the neutral condition, we used CERT to validate video recordings of her. We trimmed the footage from both conditions such that they only included the frames in which the confederate was describing the figure to the participant. In total, 10 fragments per condition (from the 10 trials of the director-matcher task) were subjected to automatic analyses in CERT. Of the AUs and prototypical emotions CERT is capable of coding, AU 6, AU 12, and the emotions of *joy* and *neutrality* were most relevant, as we would like to ascertain that the confederate conveyed different emotions in the smiling condition and in the neutral condition.

**FIGURE 4:**
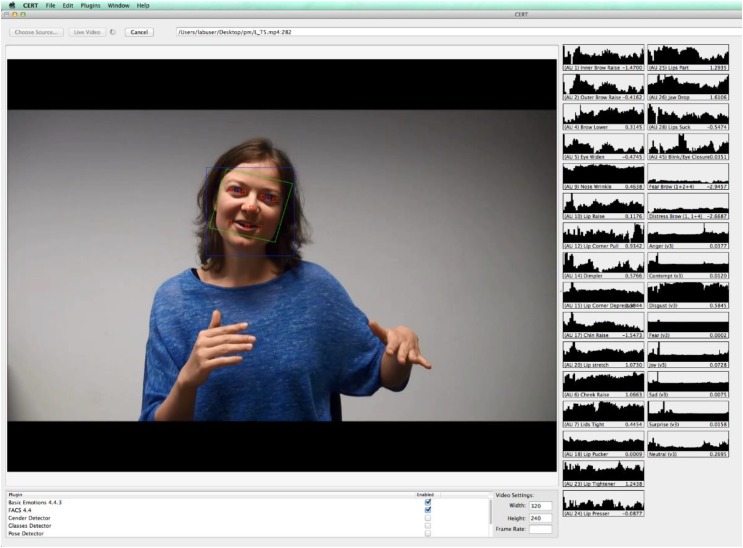
Screenshot of CERT processing the stimuli.

For each AU per frame, CERT provides a score indicating the strength or intensity of the activation. Bartlett et al. (2006) have shown that this score correlates positively with the intensity of the FACS intensity codes, and so a CERT output score can be interpreted as a shifted intensity score, i.e., large negative values represent no or little activation, and large positive values represent strong activation. For subsequent analyses, we averaged the values across frames for each fragment. A screenshot of CERT processing the stimuli is provided in Figure 4.

#### AU 6 and AU 12

The data provided by CERT were subjected to independent samples *t*-tests. A stronger activation of AU 6 was observed in the smiling condition (*M* = 1.09, *SD* = 0.07) than in the neutral condition (*M* = 0.74, *SD* = 0.09), *t*(18) = 9.80, *p* < 0.001, Cohen’s *d* = 4.38. Similarly, the activation of AU 12 was stronger in the smiling condition (*M* = 0.84, *SD* = 0.20) than in the neutral condition (*M* = -0.27, *SD* = 0.19), *t*(18) = 12.79, *p* < 0.001, Cohen’s *d* = 5.72.

#### Joy and Neutrality

In line with the results regarding AU 6 and AU 12, the presence of joy was more evident in the smiling condition (*M* = 0.31, *SD* = 0.09) than in the neutral condition (*M* = 0.07, *SD* = 0.04), *t*(18) = 7.50, *p* < 0.001, Cohen’s *d* = 3.36. Correspondingly, the expression displayed in the smiling condition (*M* = 0.08, *SD* = 0.03) was indeed less neutral than that in the neutral condition (*M* = 0.21, *SD* = 0.06), *t*(18) = 6.12, *p* < 0.001, Cohen’s *d* = 2.73. Altogether, these results and the large effect sizes demonstrated that the expressions and emotions displayed by the confederate were consistent with the experimental conditions, validating the experimental stimuli.

### Smile Mimicry

Participants were videotaped the moment the webcam was switched on; therefore, the footage also consisted of frames in which the participants were not interacting with the confederate (e.g., when the confederate moved her chair forward to sit closer to the webcam). In view of this, we analyzed a sub-set of the footage from the introductory phase and the full footage from the director-matcher task. Specifically, we analyzed 10 s of footage from the introductory phase that consisted of participant’s reactions to being introduced to the confederate and being waved at by the confederate, and the full footage of all ten trials of the director-matcher task.

Analogous to the stimuli, video recordings of the participants were subjected to automatic analyses in CERT with a focus on activity in AU 6 and AU 12. The footage of participants analyzed was characterized by not only the condition they were assigned (i.e., smiling or neutral), but also the phase from which the footage was obtained (i.e., introduction or director-matcher task). Phase was analyzed as a within-subject factor where the expressions displayed during the introduction served as a baseline; any difference in expressions displayed between the two conditions in the director-matcher task was independent of individual differences in expressive behavior. From the raw CERT output, we computed four mean values per participant, corresponding to the two AUs and the two phases of interaction. These values were used in subsequent analyses.

#### Descriptives

Means and deviations of activation in AU 6 and AU 12 are listed in Table 1 as a function of condition and phase. Overall, the activation of AU 6 ranged from -0.16 to 2.15 (*M* = 0.76, *SD* = 0.52); that of AU 12 ranged from -1.62 to 3.28 (*M* = 0.82, *SD* = 1.17).

**Table 1:** Means and standard deviations of activation in AU 6 and AU 12 of participants, as a function of interaction phase and experimental condition.

		AU 6		AU 12
		*M* (*SD*)		*M* (*SD*)
Phase				
Introduction		1.18 (0.37)		1.73 (0.76)
Task		0.33 (0.22)		-0.09 (0.69)
Condition				
Smiling (*n* = 27)		0.86 (0.54)		0.95 (1.16)
Neutral (*n* = 25)		0.65 (0.49)		0.67 (1.17)
Phase × Condition				
Introduction				
Smiling		1.31 (0.35)		1.84 (0.71)
Neutral		1.05 (0.34)		1.60 (0.81)
Task				
Smiling		0.41 (0.22)		0.06 (0.76)
Neutral		0.24 (0.20)		-0.26 (0.58)

#### AU 6

A 2 (smiling or neutral) by 2 (introduction or director-matcher task) mixed analysis of variance (ANOVA) was conducted on activation of AU 6. This revealed a main effect of phase, *F*(1,50) = 301.36, *p* < 0.001, η^2^ = 0.86: Activation of AU 6 was more intense during the introduction (*M* = 1.18, *SE* = 0.05) than during the director-matcher task (*M* = 0.33, *SE* = 0.03). Also, a main effect of condition emerged, *F*(1,50) = 11.98, *p* = 0.001, η^2^ = 0.19. AU 6 was more active among participants in the smiling condition (*M* = 0.86, *SE* = 0.04) than among participants in the neutral condition (*M* = 0.65, *SE* = 0.05). No significant interaction between phase and condition was found, *F*(1,50) = 0.67, *p* = 0.42, η^2^ = 0.01.

#### AU 12

A similar analysis on the activation of AU 12 revealed a main effect of phase, *F*(1,50) = 253.00, *p* < 0.001, η^2^ = 0.84. AU 12 was more active during the introduction (*M* = 1.72, *SE* = 0.11) than during the task (*M* = -0.10, *SE* = 0.10). While no significant effect of condition was observed, *F*(1,50) = 2.89, *p* = 0.10, η^2^ = 0.06, the estimated marginal means suggested that AU 12 was more active in the smiling condition (*M* = 0.95, *SE* = 0.11) than in the neutral condition (*M* = 0.67, *SE* = 0.12). No interaction effect was found, *F*(1,50) = 0.09, *p* = 0.77, η^2^ = 0.00.

Interestingly, these analyses showed that smiles were more intense in the introduction than in the director-matcher task, which might be related to the characteristics of the phase: Participants smiled more intensely in an initial social encounter than in the middle of a task requiring cognitive effort. Moreover, the activation of AU 6 was more pronounced among participants in the smiling condition (than those in the neutral condition). As AU 6 has been widely regarded as the marker of genuine smiles, these findings suggested smile mimicry: When interacting with a confederate beaming with joy, participants displayed smiles that were more genuine, compared to participants who interacted with a neutral confederate.

### Emotional Contagion

Reliability and descriptive statistics of the self-report measures are listed in Table 2. To investigate if emotional contagion took place, a 2 by 2 mixed ANOVA was conducted on the *joviality* ratings. Participants were more jovial after the CMC (*M* = 4.84, *SE* = 0.10) than before the CMC (*M* = 4.61, *SE* = 0.10), *F*(1,50) = 8.65, *p* = 0.005, η^2^ = 0.15. The level of joviality in the smiling condition (*M* = 4.75, *SE* = 0.13) was slightly higher than that in the neutral condition (*M* = 4.70, *SE* = 0.13), but not significantly so, *F*(1,50) = 0.07, *p* = 0.80, η^2^ = 0.00. Similarly, the interaction between phase and condition was just shy of being significant, *F*(1,50) = 3.79, *p* = 0.057, η^2^ = 0.07. A comparison of the estimated marginal means within each condition revealed that in the smiling condition, the level of joviality after the CMC (*M* = 4.94, *SE* = 0.14) was significantly higher than that before the CMC (*M* = 4.55, *SE* = 0.14), *M*_difference_ = 0.39, *SE*_difference_ = 0.11, *p* = 0.001, but this was not the case in the neutral condition (after: *M* = 4.74, *SE* = 0.15; before: *M* = 4.66, *SE* = 0.14), *M*_difference_ = 0.08, *SE*_difference_ = 0.12, *p* = 0.49. Overall, the findings suggested emotional contagion; however, they were merely indicative, as the interaction failed to reach significance.

**Table 2:** Cronbach’s alpha, means, and standard deviations of self-report measures, as a function of experimental condition.

	Before CMC		After CMC	
	α	*M* (*SD*)	α	*M* (*SD*)
Joviality (8 items)				
Smiling condition	0.89	4.55 (0.76)	0.89	4.94 (0.78)
Neutral condition	0.88	4.66 (0.68)	0.80	4.74 (0.67)
Overall	0.89	4.60 (0.72)	0.86	4.85 (0.73)
Attentiveness (4 items)				
Smiling condition	0.70	5.06 (0.58)	0.93	5.30 (0.91)
Neutral condition	0.78	4.79 (0.75)	0.74	5.09 (0.80)
Overall	0.75	4.93 (0.68)	0.89	5.20 (0.86)

### Control Variables

In each trial of the director-matcher task, participants either managed or failed to identify the target figure. As they completed 10 trials, a maximum score of 10 could be achieved; a performance score was thus computed for every participant. Participants in the smiling condition (*M* = 8.59, *SD* = 2.15) and in the neutral condition (*M* = 9.24, *SD* = 0.88) performed equally well, *t*(34.98) = 1.44, *p* = 0.16, Cohen’s *d* = 0.40. This suggested that differences in the conditions, as revealed by the CERT analyses and the joviality ratings, could not be attributed to differences in task performance.

Before and after the CMC, items of *attentiveness* were administered as fillers; however, they could also give an impression of whether there was a difference in the level of attentiveness between the two conditions. A 2 by 2 mixed ANOVA showed that participants were more attentive after (*M* = 5.19, *SE* = 0.12) than before the CMC (*M* = 4.93, *SE* = 0.09), *F*(1,50) = 5.67, *p* = 0.02, η^2^ = 0.10. Those in the smiling condition (*M* = 5.18, *SE* = 0.13) and those in the neutral condition (*M* = 4.94, *SE* = 0.13) were equally attentive, *F*(1,50) = 1.75, *p* = 0.19, η^2^ = 0.03. No interaction between the factors was observed, *F*(1,50) = 0.09, *p* = 0.76, η^2^ = 0.00. The absence of an effect of condition or an interaction effect implied that any difference between the conditions was not due to differences in attentiveness.

## Discussion

Smile mimicry and emotional contagion have been observed in studies in which participants passively viewed successive emotional stimuli and in studies based on EMG data. The diffusion of emotions has been documented in studies on text-based CMC as well. In this study, by means of an experimental paradigm simulating naturalistic audio-visual CMC, we investigated the occurrences of (1) smile mimicry and (2) emotional contagion.

We expected that participants would mimic the expressions displayed by their interaction partner during cue-rich, audio-visual CMC (Hypothesis 1). Stronger activation of AU 6 (as measured by CERT), often considered a marker of genuine smiles, was observed from participants who interacted with a smiling partner, compared to participants who interacted with a partner with a neutral expression. This finding is indicative of smile mimicry and is consistent with existing knowledge from EMG studies in which participants were passively exposed to mostly static stimuli. Alternative explanations for the finding, such as participants’ task performance and level of attentiveness, have been ruled out as well. To the best of our knowledge, it is also the first demonstration of smile mimicry in a naturalistic interaction conducted on an audio-visual CMC platform.

Given that the confederate displayed stronger activation of both AU 6 and AU 12 in the smiling condition than in the neutral condition, the absence of an effect of condition on the activation of AU 12 is perhaps surprising, given that the cell means do indicate a difference in the expected direction. The non-significant differences for AU 12 may have been due to the nature of the director-matcher task: As participants had to rely on the confederate for identifying the target figure, they may have regarded the task as a cooperative task; therefore, they may have been motivated to affiliate with the confederate, even in the neutral condition. Thus, while participants in the neutral condition completed the task with a non-smiling confederate, their motivation to affiliate may have propelled them to activate AU 12 nonetheless. This possibility is in line with the notion that AU 12 is largely under volitional control, much more so than AU 6 (e.g., Ekman and Friesen, 1982; Ekman et al., 1990).

In addition, we investigated whether emotional contagion would take place in audio-visual CMC (Hypothesis 2). Even though the mean values suggested that only participants who had interacted with a smiling partner were more jovial after the CMC, the overall effect was not significant. Hence, no conclusive evidence for emotional contagion has been observed.. It is worth noting that this finding contrasts with those of previous studies on text-based CMC, which presented strong evidence for the diffusion of emotions based on the valence of the words found in Facebook and Twitter posts (Coviello et al., 2014; Kramer et al., 2014; Ferrara and Yang, 2015).

One possible explanation for the non-significant finding regarding emotional contagion concerns the director-matcher task. As reported earlier, participants in both conditions did extremely well at identifying the target figures. By the time the task was over, participants may have been very pleased about the collaboration and their own performance. Subsequently, they may have indicated that they were jovial, regardless of how their interaction partner came across. In future studies, researchers interested in the phenomenon of emotional contagion may wish to include tasks of varying levels of difficulty and compare participants’ measures of joviality across those levels. Doing so could clarify whether the affect reported by participants was due to the experimental manipulation or the task itself. In addition to administering Likert-type measures, it could be insightful to ask participants to provide written accounts of their affect, as it might allow researchers to pinpoint the antecedent of the emotions experienced by participants as well.

### Caveats and Limitations

To the best of our knowledge, this study is one of the first to examine mimicry and emotional contagion in cue-rich, audio-visual CMC. The majority of relevant studies have either focused on text-based CMC or studied highly standardized artificial settings devoid of a communicative element. Neither approach truly reflects the kind of modern-day communication many experience on a daily basis. By contrast, we have managed to present the same emotional stimuli to all participants, and to subject participants to an environment that still bears close resemblance to actual social interactions at the same time. The fact that only two participants questioned the authenticity of the CMC they experienced, and the finding that participants remained attentive at the end of the experiment, are also testimony to the effectiveness of the paradigm.

Nevertheless, a few caveats and limitations remain. First, the nature of the director-matcher task in the present study might have facilitated the mimicry of smiles. As speculated earlier, participants may have considered the task a cooperative task. Mimicry occurs more frequently when people are motivated to affiliate with others than when they are not (Lakin and Chartrand, 2003). Had the task been competitive in nature, a different pattern of results may have been observed. Additionally, the strength of facial mimicry may be moderated by several other factors, such as ethnic group membership (i.e., whether the expresser and the perceiver are of the same ethnicity; Mondillon et al., 2007) and perceiver’s pre-existing attitude toward the expresser (Likowski et al., 2008). Including these potential moderators in future studies may provide more nuanced findings regarding the mimicry of smiles in audio-visual CMC.

Second, our work concerns the contagion of only one type of positive affect in CMC, similar to most existing works on emotional contagion and mimicry. Very few have investigated other emotions, with the exception of anger (see Hess and Fischer, 2013, for a review) or general negative affect (Coviello et al., 2014; Kramer et al., 2014; Ferrara and Yang, 2015). This is unfortunate, considering that in both online and offline exchanges, communication is not limited to such basic emotions as happiness or anger. For example, self-disclosure, a salient element of online communication (Joinson and Paine, 2007) that has been shown to occur more frequently in CMC than in face-to-face interactions (Jiang et al., 2013), entails a myriad of feelings and experiences that cannot be characterized by happiness or anger alone. When discussing such personal issues, people may feel guilty, proud, or ashamed, all of which are self-conscious emotions (Tangney, 1999; Tracy and Robins, 2004) distinct from basic emotions. As some of these non-basic emotions have recognizable non-verbal displays, studying a wider range of emotions would offer insights into the robustness of the phenomenon of facial mimicry and emotional contagion.

Third, by limiting our analyses to the average level of smile intensity per participant by condition, we were not able to examine the role of timing in mimicry. In dyadic interactions, changes in facial expressions displayed by the two interactants over the course of an interaction can be treated as two separate time series (e.g., Griffin et al., 2015; Varni et al., 2017). Coupling these time series can allow for an observation of the time elapsed between the onset of one’s facial expression and the onset of the interactant’s facial expression. In others words, a more precise distinction between mimicry and non-mimicry is made possible. For example, if one were to start smiling only 30 s after one’s interactant had begun smiling, it would seem unlikely that such behavior was a case of mimicry. Considering the dynamic and social nature of smile mimicry, timing is an important factor that should be taken into account in future studies.

We opted for a comparison of two experimental conditions within audio-visual CMC, rather than a comparison with another medium. Whilst it may seem reasonable to introduce another medium (e.g., face-to-face interaction) into our study, we did not do so for both theoretical and methodological reasons. Our decision is in line with previous text-based CMC studies on the diffusion of emotions, in which comparisons were also made between different conditions in only one form of CMC. In terms of methodology, a key strength of our study is the stringent experimental control it allows for: We could present the same dynamic stimuli to all participants assigned to the same condition, by virtue of the stimuli being pre-recorded footage of the confederate. This could not be achieved in a face-to-face setting in a way that would make it comparable to the audio-visual CMC setting. As this study focuses on smile mimicry, the specific facial expressions displayed by the confederate are crucial. We find it unlikely that in an actual face-to-face interaction, a confederate is capable of displaying specific facial expressions at timed intervals in an identical manner to all participants, especially considering that the full interaction is fairly long. Hence, given the goal and scope of this study, we believe that focusing on audio-visual CMC only is a sound choice.

## Conclusion

In this study, we set out to investigate smile mimicry and emotional contagion in the context of non-text-based online communication. By means of a paradigm simulating naturalistic audio-visual CMC, we have shown that people interacting with a smiling partner displayed more genuine smiles than those interacting with an unexpressive partner. However, participants who had interacted with a smiling partner reported experiencing just as much joviality as participants who had interacted with an unexpressive partner. In sum, our findings suggest that audio-visual CMC is a medium via which smiles can be successfully transmitted and mimicked, but that the evidence for emotional contagion is not as conclusive. We believe more investigations are necessary for establishing a more solid understanding of the contagion of emotions in audio-visual CMC.

## Author Contributions

PM, CR, WS, and MS contributed to the design of the study. WS performed data collection. CR and WS wrote early drafts of sections of the manuscript. PM wrote the full manuscript. MG and MS contributed to manuscript revision and supervised the whole process. All authors were involved in the statistical analyses and contributed to the manuscript, read, and approved the submitted version.

## Conflict of Interest Statement

The authors declare that the research was conducted in the absence of any commercial or financial relationships that could be construed as a potential conflict of interest.
